# Blood Flow–restricted Exercise Does Not Induce a Cross-Transfer of Effect: A Randomized Controlled Trial

**DOI:** 10.1249/MSS.0000000000001984

**Published:** 2019-03-19

**Authors:** KWASI AMPOMAH, SHINICHI AMANO, NATHAN P. WAGES, LAUREN VOLZ, RACHEL CLIFT, ARIMI FITRI MAT LUDIN, MASATO NAKAZAWA, TIMOTHY D. LAW, TODD M. MANINI, JAMES S. THOMAS, DAVID W. RUSS, BRIAN C. CLARK

**Affiliations:** 1 Ohio Musculoskeletal and Neurological Institute (OMNI), Ohio University, Athens, OH; 2 Clinical and Translational Research Unit (CTRU), Ohio University, Athens, OH; 3 Faculty of Health Sciences, Universiti Kebangsaan Malaysia, Kuala Lumpur, MALAYSIA; 4 Office of Research and Grants, Ohio University Heritage College of Osteopathic Medicine, Athens, OH; 5 Department of Family Medicine, Ohio University, Athens, OH; 6 Institute on Aging and the Department of Geriatric Medicine, University of Florida, Gainesville, FL; 7 Division of Physical Therapy, the School of Rehabilitation and Communication Sciences, Ohio University, Athens, OH; 8 Department of Biomedical Sciences, Ohio University, Athens, OH

**Keywords:** KAATSU, TRUNK EXTENSOR, MUSCLE STRENGTH, MUSCLE MASS

## Abstract

**Purpose:**

The goal of this trial was to determine whether low-load blood flow–restricted (BFR) exercise of appendicular muscles induces a cross-transfer of effect to the trunk extensor (TE) muscles, such that low-load TE exercise would enhance TE size and function to a greater extent than standard low-load exercise in people with recurrent low back pain (LBP). We also investigated the direct effects of BFR exercise in the appendicular muscles.

**Methods:**

Thirty-two adults with recurrent, nonspecific LBP were randomized into two groups: Appendicular BFR exercise (BFR exercise) or control exercise (CON exercise). All participants trained (two times per week) for 10 wk, with a 12-wk follow-up. Participants performed three sets of leg extension (LE), plantar flexion (PF), and elbow flexion (EF) exercises followed by low-load TE exercise without BFR. Outcome measures included magnetic resonance imaging–derived muscle size (quadriceps and TE), strength (LE, PF, EF, and TE), and endurance (LE and TE).

**Results:**

There was no evidence for a cross-transfer of effect to the TE. There was also no statistically significant enhancement of limb skeletal muscle size or function of BFR relative to CON exercise at any time point; though, moderate effect sizes for BFR exercise were observed for enhanced muscle size and strength in the leg extensors.

**Conclusions:**

Low-load BFR exercise of the appendicular muscles did not result in a cross-transfer of effect to the TE musculature. There was also no significant benefit of low-load BFR exercise on the appendicular muscle size and function, suggesting no benefit from low-load BFR exercise in adults with recurrent, nonspecific LBP.

## Abstract 2

Supplemental digital content is available in the text.

More than 25 yr ago Sundberg and colleagues (1) suggested that performing exercise under conditions of modest blood flow restriction (BFR) (referred to in their seminal article as “ischemic training”) resulted in enhanced skeletal muscle adaptations compared with standard exercise. Over the past several decades, there has been growing interest in the potential for low-load BFR exercise, also referred to as Kaatsu exercise, to enhance skeletal muscle adaptations to exercise (e.g., enhanced muscle mass, strength, endurance, etc.). Indeed, a number of studies have noted that low-load BFR exercise can serve as a potent stimulus for increases in muscle size, strength, and endurance, relative to exercise without BFR (for reviews and meta-analyses see (2–7)). As such, BFR exercise has gained popularity as a novel exercise modality in both performance and rehabilitation settings.

In addition to the reported effects of BFR exercise listed above, there is evidence that BFR exercise might promote hypertrophy in muscles not exercising under “traditional” BFR conditions via *systemic* hypertrophy-promoting effects (8–11). For instance, Madarame and colleagues reported an increase in muscle size (12%) and strength (9.8%) of the elbow flexors after 10 wk of low-load resistance exercise coupled with BFR of the legs, but not arms (8). This purported phenomenon, commonly referred to as a “cross-transfer of effect,” has been reported in several studies (8–11). The putative mechanism for this effect is increased systemic endocrine growth factors after BFR exercise (12–16). For instance, prior work suggests that resistance training with BFR amplifies normal exercise-induced high-energy phosphate depletion and muscle pH reduction, and maintains this altered metabolic milieu (6). A strong stimulation of the metaboreflex by these conditions could account for findings from numerous studies reporting that single bouts of BFR exercise increase serum growth hormone and IGF-1 levels (12–16) to levels comparable to, or greater than, those observed during resistance training at much higher intensities (17). However, this underlying scientific premise has also been called into question by data suggesting no systemic hormonal influence on hypertrophy or strength gains (18,19). Accordingly, the overarching goal of this work was to rigorously determine whether a “cross transfer of effect” occurs in association with BFR resistance exercise. We chose to investigate the potential phenomenon within the context of the trunk extension musculature in a nonspecific low back pain (LBP) population. The potential to apply the cross-transfer of BFR to the trunk muscles could have important therapeutic benefit in nonspecific LBP.

Although the pathogenesis of nonspecific LBP remains unknown, weakness and fatigability of the trunk extensor (TE) muscles are predictive of first time episodes of LBP, as well as recurrence (20–22). Dramatic atrophy of the lumbar multifidus muscle occurs after experimental disc and nerve root injuries (23), and patients with both recurrent and chronic nonspecific LBP exhibit wasting of the TE (24–29). Collectively, these findings indicate that patients with nonspecific LBP, particularly recurrent, nonspecific LBP, exhibit deconditioned TE muscles (30). The vast majority of TE exercises for the rehabilitation of nonspecific LBP are performed at low loads (31), mostly out of concern over the high mechanical and compressive loading on the spine (31,32). There is a need to develop therapeutic exercise paradigms to induce adaptation in the TE musculature without high-mechanical and compressive loading on the spine. Thus, if BFR exercise of limb muscles were to induce a cross-transfer effect to the TE, the clinical significance and impact could be high. The, present study was to designed as a preliminary trial to determine whether low-load BFR exercise of appendicular muscles induces a cross-transfer of effect to the TE muscles, such that low-load TE exercise would enhance TE size and function to a greater extent than standard low-load exercise in people with recurrent, nonspecific LBP. Accordingly, randomized assignment and blinding of study personnel to treatment group assignment were applied throughout the study. In addition, individuals in both exercise groups performed the appendicular exercises to volitional failure (to control for potential bias in the study design—see discussion for further comment). In doing so, we were also able to investigate the *direct effects* of BFR exercise in the appendicular muscles, which is impactful when one considers that many studies on BFR exercise have been criticized for being lower-quality studies (3).

## METHODS

### Study Design

This study was a single-blinded, single-site, randomized controlled trial, using a two-by-three (group–time) repeated-measures factorial design. The Ohio University Institutional Review Board approved this study, and written informed consent was obtained from each individual before participating. This study protocol has previously been published in detail (33) and is registered on ClincialTrials.gov (NCT02308189). Subject recruitment began in December 2014, and the primary completion date of the study was November 2016. Participants were randomized, in a 1:1 ratio, into one of two groups (see CONSORT Diagram, Supplemental Digital Content 1, The Consolidated Standards of Reporting Trials (CONSORT) Flow Diagram, http://links.lww.com/MSS/B568). Group 1 received 10 wk of resistance exercise training with BFR (BFR exercise group), whereas group 2 received 10 wk of resistance exercise training without BFR (CON exercise group). Randomization was stratified by sex, using a permuted-block design, for the purpose of maintaining balance across treatment groups. Study recruitment and eligibility testing, as well as randomization of subjects were performed by a project manager of the Ohio University Clinical and Translational Research Unit. Careful investigator bias control measures included: 1) study personnel conducting the outcome assessments (including image analysis) being blinded to group assignment throughout the length of the study, and 2) the statistician (M.N.) and principal investigator (B.C.) remaining blinded to group assignment throughout the study (they were unblinded after the data and statistical analyses were completed).

The chronological sequence of this study consisted of baseline testing, a 10-wk exercise training intervention, and a 12-wk follow-up period. Specifically, outcome measures were assessed at baseline, immediately after the completion of the exercise intervention (primary endpoint), and at week 12 of the follow-up period (secondary endpoint). Subjects were also asked to maintain their typical daily lifestyle (e.g., activity, diet, sleep patterns, etc.) throughout the course of the study to minimize confounding variability. Additionally, subjects were instructed to report for all outcome measure testing sessions in a well-hydrated state.

### Subjects

Thirty-two young adult subjects with recurrent, nonspecific LBP were randomly assigned to an exercise control (ExCon; *n* = 16) or BFR exercise (ExBFR; *n* = 14) group (see CONSORT Diagram, Supplemental Digital Content 1, The Consolidated Standards of Reporting Trials (CONSORT) Flow Diagram, http://links.lww.com/MSS/B568). Table 1 describes the inclusion and exclusion criteria in detail. To be eligible for participation, individuals had to be between 18 and 50 yr with recurrent, nonspecific LBP. We operationally defined recurrent, nonspecific LBP as individuals who answer “yes” to the following question: Have you had two or more episodes of LBP in the past 12 months with at least one episode causing a restriction of work or leisure time activity? A further criterion was poor TE muscle endurance (<176 s on a modified Sorenson test) an isometric trunk extension endurance test. In a prospective study a mean time of 176 s was noted in those who went on to develop LBP as opposed to a mean time of 198 for those who did not (20). Individuals who had participated in progressive resistance exercise, within the previous 24 wk (before screening) were also excluded. The criteria were designed to recruit a population with recurrent, nonspecific LBP, but to exclude potential participants who are currently experiencing a level of LBP above where we had concerns about the pain interfering with a participant’s ability to appropriately perform the exercise prescription (e.g., exclusion criteria resulting in the exclusion of potential participants whose current pain level is greater than 4 on a 0–10 scale).

**TABLE 1:**
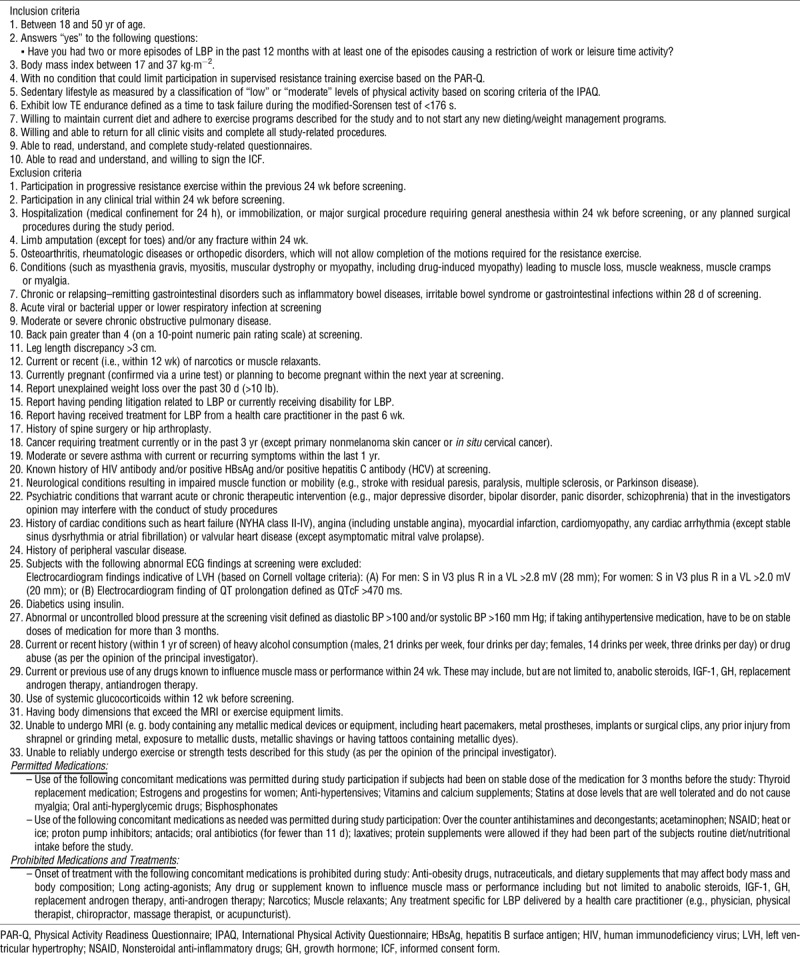
Inclusion and exclusion criteria.

### Outcome Measures

Study outcomes were assessed at the Ohio Musculoskeletal and Neurologic Institute at Ohio University (Athens, OH). Outcome measures were: 1) quadriceps femoris and lumbar erector spinae muscle cross-sectional area (CSA) measurements derived via magnetic resonance imaging (MRI), 2) voluntary isometric muscle strength of the leg extensors, elbow flexors, plantar flexors, and TE, 3) leg extensor and TE isometric muscle endurance, 4) LBP, and 5) treatment acceptability. Methodological details are presented below.

#### Muscle CSA

Magnetic resonance imagings were obtained using a 0.25-T Musculoskeletal MRI system (Esaote G-Scan Brio, Genoa, Italy). We acquired contiguous transverse T-1 weighted spin echo image slices in the trunk region between L2 and L5 and then, in a subsequent scan, the mid-thigh, with a slice thickness of 10 mm for both scans. To ensure the consistency within/among subjects, the isocenter was positioned at the midpoint of the L3/L4 intervertebral disc and one third of the distance between the knee and the greater trochanter, respectively. Before all scans, subjects rested supine for at least 15 min to minimize the effects of fluid shifts on volumetric calculations. The posttesting MRI scan was obtained 3 d after the final training session to minimize the effects of exercise-induced fluid shifts. Scanned images were transferred to a computer for calculation of muscle anatomical CSA for the erector spinae and quadriceps femoris muscle groups (an average of three slices at the isocenter from both sides) using the Medical Image Processing, Analysis, and Visualization image analysis software. Extreme care was taken to insure that the slices chosen for analysis were of the same anatomical location at all time points (accomplished by matching the slices for anatomical features [i.e., fascial characteristics]). Intermuscular fat was subtracted from the calculations based on pixel intensity as we have previously described (34,35).

#### Muscle strength

Voluntary muscle strength was quantified for the leg extensors, elbow flexors, plantar flexors, and the TE. For the leg extensors participants were seated in a MedX leg extension dynamometer (Ocala, FL), which allowed for strict control of hip and knee joint angles. The backrest was adjusted so subjects sat with a hip joint angle of 100° from flexion, and a seat belt was secured to prevent any movement of the hip joint. The limbs were attached to the force transducer (model U1T; HBM Inc., Marlborough, MA), and the knee joint angle was set at 60° from extension. For the elbow flexors subjects wore an arm isolator (Rogue Arm Blaster; Rogue Fitness, Columbus, OH) and stood with their feet shoulder width apart with the arms attached to a force transducer (TSD121C; BioPac Systems Inc. Santa Barbara, CA) with the elbows positioned at 90°. For the plantar flexors, subjects were seated in a custom-modified dynamometer with the legs positioned in the dynamometer with the hip, knee, and ankle joint angles all secured at 90°, and force was measured by a force transducer (TSD121C; BioPac Systems). For the TE, subjects were seated in a lumbar extension dynamometer (MedX, Ocala, FL) with the upper body positioned in the upright, neutral position, and force was measured via a force transducer (model U1T; HBM Inc.). Femur and lap restraints were applied to stabilize the pelvis. During the strength assessments, the exerted force was displayed on a computer monitor displayed in front of the subject. All force signals were amplified and recorded at 500 Hz using a 16-bit data acquisition card (MP150; BioPac Systems Inc.). A minimum of three trials for each task were performed, with additional trials provided as needed if subjects continually exert more force with each trial or if the highest two trials were not within 5%. Each contraction lasted approximately 5 s with at least a 60-s rest period. Muscle strength was defined as the highest value recorded in any trial.

#### Muscle endurance

The time to task failure of a sustained, submaximal isometric contraction of the leg extensors and the TE were determined (mechanical setup the same as described for the strength assessment with the exception that the leg extension task was performed unilaterally with the nondominant limb). During these tests, participants performed the tasks at 20% of their baseline muscle strength by matching a target line on a computer monitor until volitional task failure similar to our previous descriptions (36,37). During the tasks, a target line was displayed on a computer monitor placed in front of the participant and the time to task failure will be quantified. Task failure was determined to occur when the feedback line drifted below the target force line by 5% for longer than 3 s despite verbal encouragement being provided to subjects to restore the position of the force line if they drifted away from the target force.

#### Treatment acceptability

Treatment acceptability was determined by administering the Treatment Evaluation Inventory Short Form survey at the end of every fourth exercise session (38).

### Exercise Interventions

Supervised exercise training sessions were conducted twice per week for 10 wk. For both training groups the exercise intensity was set at 25% of their maximal voluntary isometric strength. Our rationale for choosing an intensity of 25% is that it is similar to many studies on low-load exercise with BFR, including our own, which have shown positive muscle adaptations with intensity levels in the range of 25% to 30% of maximal strength (8,11,39,40). To ensure that exercise intensity values were adjusted accordingly to strength progression, reassessment of maximal strength was performed during the fifth week of the training schedule. Participants in the BFR exercise group performed three sets of leg extension (MedX leg extension dynamometer), plantar flexion (seated calf raises), and elbow flexion (arm curls on cable machine) exercises to task failure while BFR was applied to the proximal limbs by a KAASTU Master device (KAATSU Training Japan Co., Ltd., Tokyo, Japan). Subjects were allowed 30 to 60 s of rest between sets. While performing leg extension and plantar flexion exercises, the pressure cuff was placed on the upper thigh, just below the gluteal fold. While performing elbow flexion exercises, the pressure cuff was placed on the upper arm, just below the shoulder joint. The cuff pressure for each limb was determined on each day of exercise for an individual. The initial cuff pressure was applied in accordance with the KAATSU protocol (41), which involved setting the baseline pressure at approximately 30 to 40 mm Hg and then applying additional pressure in increments of 20 mm Hg. This inflation–deflation sequence continued until the circulation in the limbs was impeded, but not occluded. Specifically, the cuff pressure for the leg and arm was set when the capillary refill time of the leg, just above the knee, or the palms of the hands was between 2 and 3 s (41). The pressure cuffs remained inflated until the completion of all three sets of exercise, including the rest periods. The average cuff pressures for the first exercise session were 142.9 ± 33.3 mm Hg and 160.7 ± 34.2 mm Hg for the arms and legs, respectively. The inflation pressure was increased progressively throughout the 10-wk training period based on subject tolerance and capillary refill time. The average cuff pressures for the last exercise session were 205.7 ± 48.3 mm Hg and 240.0 ± 49.8 mm Hg for the arms and legs, respectively. After completing the BFR exercises, participants in the BFR exercise group performed three sets of 15 repetitions of TE exercises at 25% of maximal voluntary isometric strength while seated in a MedX TE dynamometer. The CON exercise group performed an identical exercise protocol as the BFR exercise group, except that BFR was not applied to the appendicular limbs. An overview of the two exercise groups is illustrated in Table 2. We chose the specific exercises based on their being clinically viable and adaptable to BFR exercise.

**TABLE 2:**
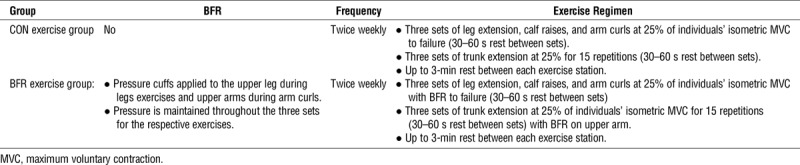
Schematic overview of the exercise intervention groups.

### Statistical Analyses

Sample size calculations for this study have been previously described (33). For the three skeletal muscle-related outcomes (i.e., muscular size, strength, and endurance), we computed a percent change score between values at pretraining and immediately after the 10-wk intervention period, and between values at pretraining and the postintervention 12-wk follow-up period. Afterward, we tested group percent differences using linear models, with covariates included to increase the power to detect a significant treatment effect, while also controlling for the potential confounding effects of the covariates (sex, standardized age [using a *z*-score transformation], standardized pretreatment value). In this analysis the group difference (which we refer to in the results as “difference”) corresponds to the test of the interaction between time and group. One participant (control group) had extreme MRI values (standardized scores greater than three SD away from the mean) and was excluded from the muscle size analyses. We subsequently identified this to be due to movement artifact in the MRI).

Next, we performed an intention-to-treat analyses for all randomized participants who had baseline assessments and estimated parameters based on maximum likelihood estimation. We also performed an exploratory per-protocol analyses, where we excluded participants who had: 1) failed to attend 75% of their exercise training sessions, 2) received prohibited concomitant interventions, or 3) developed an exclusionary medical condition while on study protocol. All data were expressed as mean ± SD unless otherwise noted (error bars in figures represent 95% confidence interval). We performed all statistical tests at the 5% significance level (two-tailed), and because this was a proof-of-concept trial (i.e., exploratory in nature) we did not adjust the α level for multiple comparisons. To further aid in the interpretation of data we also report effect sizes (eta-squared [*η*^2^]) at the primary endpoint.

We also ran an analysis of covariance with group and time entered into the model with baseline values as covariates. The findings from this analysis were congruent with the percent change analysis described above. We chose to present the percent change results for simplicity and reader clarity. Unpaired *t*-tests were used to examine group differences at baseline.

### Adverse Events

At each study-related visit we carefully documented any changes in health status. Adverse events (AE) were documented and assessed for severity (grade) and attribution by the project’s medical director (T.L.) according to the National Cancer Institute’s Common Terminology Criteria for Adverse Events (CTCAE) guidelines (42). In brief, the CTCAE displays grades 1 through 5 with unique clinical descriptions of severity for each AE based on this general guideline:

- Grade 1, mild: asymptomatic or mild symptoms; clinical or diagnostic observations only; intervention not indicated.
- Grade 2, moderate: minimal, local, or noninvasive intervention indicated; limiting age-appropriate instrumental activity of daily living.
- Grade 3, severe or medically significant but not immediately life-threatening; hospitalization or prolongation of hospitalization indicated; disabling; limiting self-care activity of daily living.
- Grade 4, life-threatening consequences; urgent intervention indicated.
- Grade 5, death related to AE.

The classification of potential relationship to the intervention (i.e., attribution) was as follows:

- Definite: temporal pattern + known or expected AE response pattern + Confirmed by stopping the intervention + reappearance of AE on rechallenge.
- Probable: temporal pattern + known or expected AE response pattern + confirmed by stopping the intervention + could not be explained by participant’s clinical state.
- Possible: temporal pattern + known or expected AE response pattern + could have been produced by a number of other factors.
- Unknown: relationship for which no evaluation can be made.
- Not related: AE for which sufficient information exists to indicate that the cause is unrelated to the study intervention.

## RESULTS

A total of 574 individuals were screened for this study, and of those individuals screened, 35 met the eligibility criteria. Three chose not to participate, leaving 32 to be randomized in the study. Fifteen participants were randomized to the BFR exercise group (10 females and 5 males), and 17 participants were randomized to the CON exercise group (10 females and 7 males). Two participants were withdrawn from the study after the randomization process (*n* = 1 female/group). The first was withdrawn due to the baseline strength testing session exacerbating their LBP (CON exercise group). The second was withdrawn during the follow-up period after they reported seeking physical therapy after the completion of the study exercise protocol (BFR exercise group). Thus results were analyzed for 14 participants in the BFR exercise group and 16 participants in the CON exercise group. Baseline characteristics of the subjects are detailed in Table 3 (no significant group differences were observed on any of the characteristics).

**TABLE 3:**
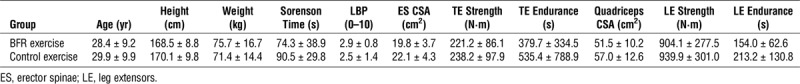
Descriptive statistics of the study participants at baseline.

### Cross-Transfer Effect of BFR Limb Exercise on TE Size and Function

#### Erector spinae CSA

**FIGURE 1:**
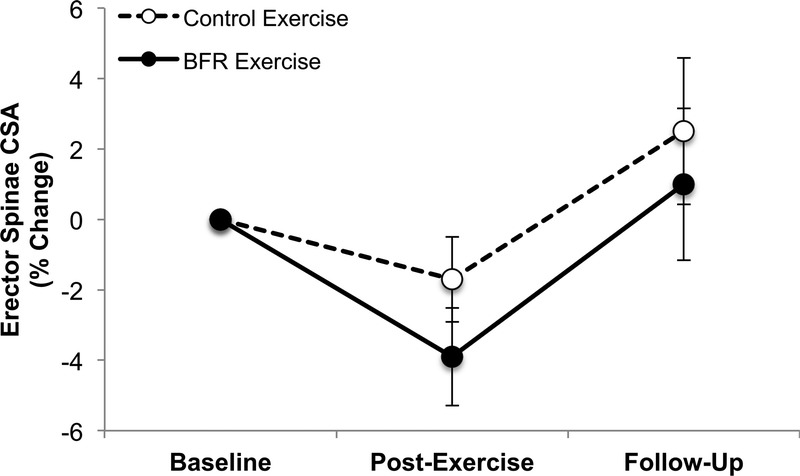
BFR exercise did not result in a significant cross-transfer of effect in erector spinae muscle CSA. Error bars represents 95% CI. 95% CI, 95% confidence interval.

At the primary endpoint, there were no significant differences within or between groups (BFR exercise group, −3.9% ± 2.7%, *P* = 0.2; CON exercise group, −1.7% ± 2.5%, *P* = 0.5; group difference: *P* = 0.5; *η*^2^ = 0.15) (Fig. 1). At the secondary endpoint, there were again no significant differences within or between groups (BFR exercise group, 1.0% ± 4.2%, *P* = 0.8; CON exercise group, 2.5% ± 4.3%, *P* = 0.5; group difference, *P* = 0.8) (Fig. 1). When collapsed across exercise groups, no significant changes in erector spinae CSA muscles were observed at either the primary (−2.6% ± 2.1%, *P* = 0.2) or secondary (1.8% ± 3.4%, *P* = 0.6) endpoints.

#### TE strength

**FIGURE 2:**
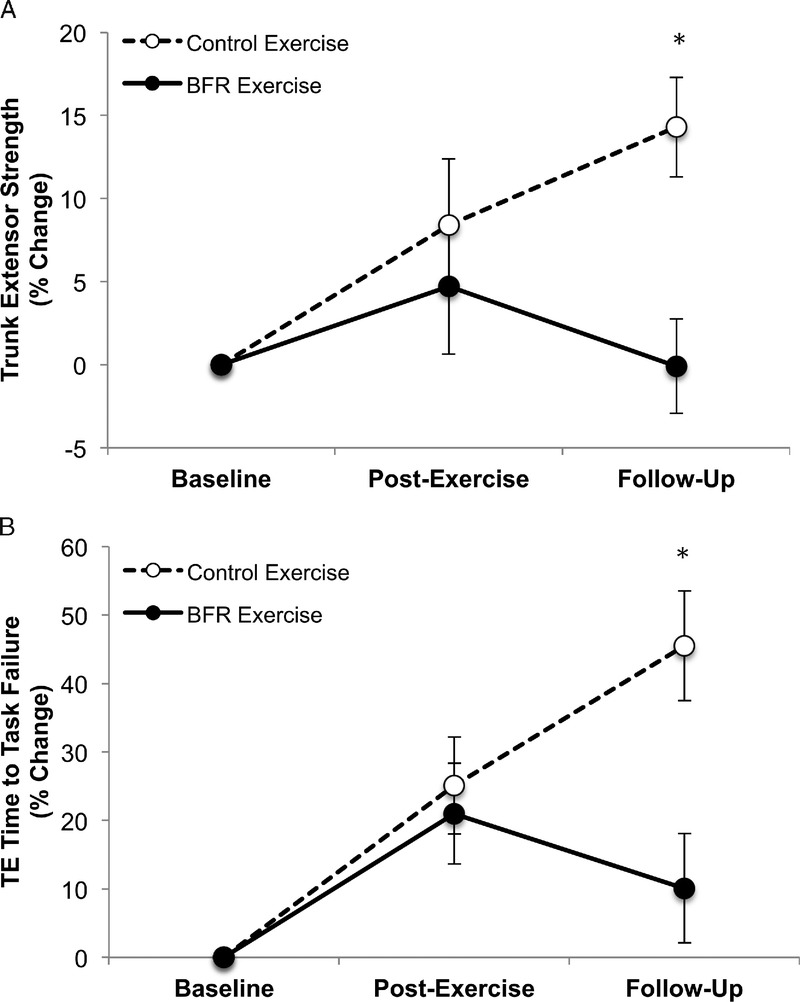
BFR exercise did not result in a significant cross-transfer of effect in TE muscle strength (A) or endurance (B) (note that beneficial effects noted in the follow-up period are in the CON exercise group). Error bars represents 95% CI.

At the primary endpoint, neither the participants in the BFR exercise group, nor the CON exercise group, exhibited a significant change in strength relative to baseline BFR (exercise group: 4.7% ± 7.9%, *P* = 0.6; CON exercise group: 8.4% ± 8.2%, *P* = 0.3) (Fig. 2A). Furthermore, there were no significant differences found between the groups (group difference, *P* = 0.7; *η*^2^ = 0.05) (Fig. 2A). In contrast, at the secondary time point, there was a significant increase in TE strength compared to baseline for the CON exercise group, but not for the BFR exercise group (BFR exercise group, −0.1% ± 5.5%, *P* = 0.9; CON exercise group, 14.3% ± 6.2%, *P* = 0.03) (Fig. 2A). The between group difference was also found to be significant (*P* = 0.04). When we collapsed the two exercise groups, TE strength was not affected by exercise at either the primary endpoint (6.4% ± 6.5%, *P* = 0.3) or secondary endpoint (5.8% ± 5.1%, *P* = 0.3).

#### TE endurance

At the primary endpoint, neither the participants in the BFR exercise group, nor the CON exercise group, exhibited a significant percent change in time to task failure (BFR exercise group, 21.0% ± 14.3%, *P* = 0.2; CON exercise group, 25.1% ± 14.6%, *P* = 0.2), nor was there a difference between the groups (*P* = 0.8, *η*^2^ = 0.03) (Fig. 2B). In contrast, at the secondary endpoint, there was a significant increase in time to task failure for the CON exercise group (45.5% ± 17.0%, *P* = 0.01), but not the BFR exercise group (10.1% ± 15.5%, *P* = 0.5); however, there were no significant differences found between the groups (*P* = 0.09) (Fig. 2B).

### Effects of BFR Limb Exercise on Appendicular Muscle Size and Function

#### Quadriceps femoris muscle CSA

**FIGURE 3:**
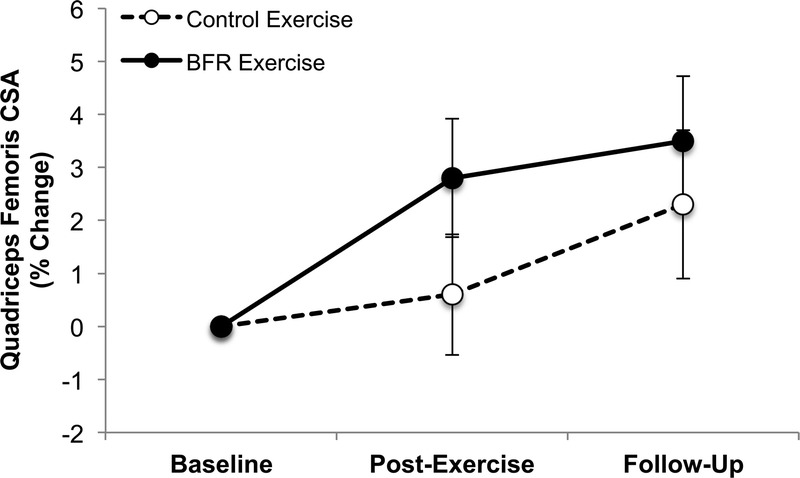
BFR exercise did not significantly increase quadriceps femoris muscle CSA. Error bars represents 95% CI.

We found no significant differences within or between groups at either time point (Fig. 3). At the primary endpoint, there were no significant differences found within or between groups (BFR exercise group, 2.8% ± 2.3%, *P* = 0.2; CON exercise group, 0.6% ± 2.2%, *P* = 0.5; group difference: *P* = 0.4); however, a moderate effect size (*η*^2^ = 0.19) was noted for the interaction term (Fig. 3). At the secondary endpoint, there were again no significant differences found within or between groups (BFR exercise group, 3.5% ± 2.5%, *P* = 0.3; CON exercise group, 2.3% ± 2.7%, *P* = 0.4; group difference: *P* = 0.7, *η*^2^ = 0.05) (Fig. 3). Due to a lack of a group effect for the MRI measure, we collapsed the two exercise groups to determine if exercise, regardless of whether BFR was administered, affected limb muscle size and function after controlling for the covariates (sex, standardized age, and standardized pretreatment value). We did not observe a significant effect of exercise on CSA measured at the primary endpoint (1.6% ± 1.8%, *P* = 0.4), or secondary endpoint (2.9% ± 2.2%, *P* = 0.19).

#### Appendicular muscle strength

**FIGURE 4:**
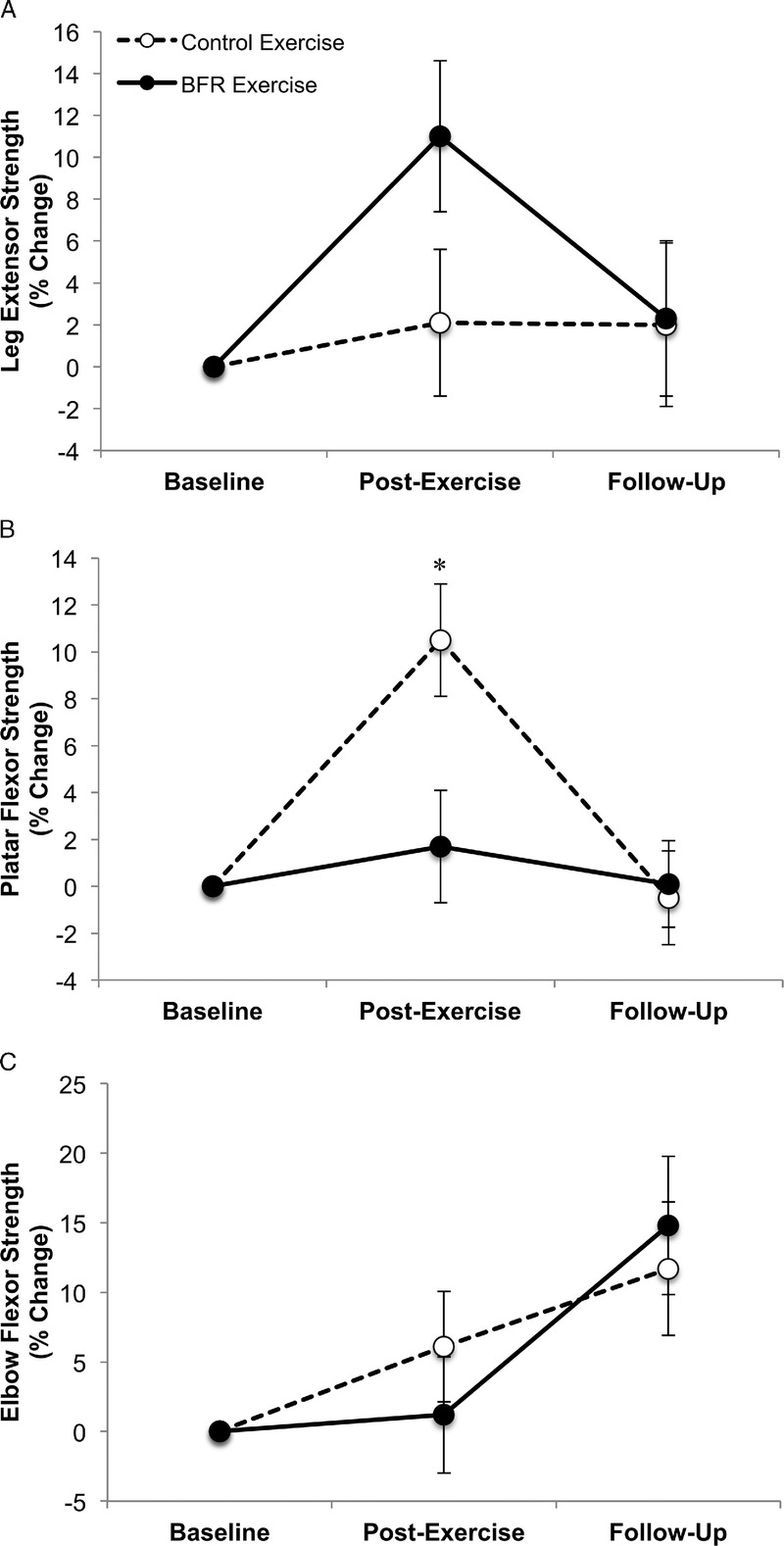
Effect of BFR exercise on appendicular muscle strength of the leg extensors (A), plantar flexors (B), and elbow flexors (C). Panel D illustrates the effect of BFR exercise on leg extensor (LE) muscle endurance. For plantar flexor strength and leg extensor endurance the control exercise group exhibited a significant increase postexercise (*plantar flexor, *P* = 0.05; leg extensor endurance, *P* = 0.04); however, the group difference was only significant for the plantar flexors (plantar flexor group difference, *P* = 0.05, *η*^2^ = 0.84; leg extensor endurance group difference, *P* = 0.2; *η*^2^ = 0.39). Error bars represents 95% CI.

There were no consistent patterns for strength changes among the three appendicular muscle groups at the primary or secondary endpoints (i.e., there was no systematic change [positive or negative] noted across the various muscle groups) (Fig. 4). At the primary and secondary endpoints, leg extensor strength did not significantly increase for either the BFR exercise group, or the exercise control group, and there were no significant differences between the groups (*Primary Endpoint*: BFR exercise group: 11.0% ± 7.1%, *P* = 0.1; CON exercise group: 2.1% ± 7.3%, *P* = 0.8; group difference: *P* = 0.3; *Secondary Endpoint*: BFR exercise group: 2.3% ± 7.2%, *P* = 0.7; CON exercise group: 2.0% ± 8.0%, *P* = 0.80; group difference: *P* = 0.9) (Fig. 4A). There was a moderate effect size for strength enhancement in the BFR exercise group (*η*^2^ = 0.27) at the primary endpoint. In contrast, plantar flexor strength significantly increased in the CON exercise group, but not the BFR exercise group at the primary endpoint (Fig. 4B), and there was a significant group difference (*Primary Endpoint*: BFR exercise group, 1.7% ± 4.7%, *P* = 0.7; CON exercise group, 10.5% ± 5.0%, *P* = 0.05; group difference: *P* = 0.05; *η*^2^ = 0.84). At the secondary endpoint, there was no significant difference found within or between groups (*Secondary Endpoint*: BFR exercise group, 0.1% ± 3.6%, *P* = 0.9; CON exercise group, −0.5% ± 4.2%, *P* = 0.9; group difference: *P* = 0.9). Finally, at the primary and secondary endpoint, elbow flexor strength did not significantly increase for either the BFR exercise group, or the CON exercise group, and there were no significant differences found between the groups (*Primary Endpoint*: BFR exercise group, 1.2% ± 8.1%, *P* = 0.9; CON exercise group, 6.1% ± 8.2%, *P* = 0.5; group difference, *P* = 0.6; *η*^2^ = 0.08; *Secondary Endpoint*: BFR exercise group, 14.8% ± 9.6%, *P* = 0.08; CON exercise group, 11.7% ± 9.9%, *P* = 0.03; group difference, *P* = 0.8) (Fig. 4C). Overall, exercise (when collapsed across exercise groups) did not significantly affect any measure of appendicular muscle strength at the primary endpoint (leg extensor: 6.8% ± 5.8%, *P* = 0.3, *η*^2^ = 0.26; plantar flexor: 5.7% ± 3.9%, *P* = 0.15, *η*^2^ = 0.35; elbow flexor: 3.6% ± 6.4%, *P* = 0.6, *η*^2^ = 0.07) or secondary endpoint (leg extensor: 2.2% ± 6.0%, *P* = 0.7, *η*^2^ = 0.03; plantar flexor: −0.1% ± 3.0%, *P* = 0.9, *η*^2^ < 0.01; elbow flexor: 13.3% ± 7.8%, *P* = 0.1, *η*^2^ = 0.42).

#### Leg extensor endurance

We further examined whether BFR exercise influenced leg extensor muscular endurance at the primary and secondary endpoints. At the primary endpoint, time to task failure significantly increased for the CON exercise group, but not for the BFR exercise group (CON exercise group, 35.2% ± 16.2%, *P* = 0.04; BFR exercise group, 9.6% ± 15.8%, *P* = 0.6). However, there were no significant differences found between the groups (*P* = 0.2), although a moderate effect size was observed for an enhancement in the CON exercise group (*η*^2^ = 0.39). At the secondary endpoint, there were no significant differences within or between groups (BFR exercise group, −2.1% ± 9.5%, *P* = 0.8; CON exercise group, 15.0% ± 11.0%, *P* = 0.19; group difference: *P* = 0.2; *η*^2^ = 0.41). When we collapsed knee extensor endurance across groups, the primary endpoint was not significant, although a moderate mean increase was observed (22.1% ± 12.4%, *P* = 0.09), while at the secondary endpoint, we did not observe a similar effect (4.9% ± 7.9%, *P* = 0.6).

### Per Protocol Analysis

In addition to the intention-to-treat analysis described above, we also performed a per protocol analysis where we identified and excluded participants who failed to attend 75% of the exercise training sessions. This resulted in one male participant, from the CON exercise group, being excluded. After removing this participant, all analyses were repeated and found that excluding this participant did not change the significance or interpretation of any of the results described above.

### AE and Treatment Acceptability

There were a total of nine AE reported in eight subjects. None of the AE were deemed serious (five mild and four moderate). Seven of the AE were deemed clearly unrelated to the study protocol. Table 4 summarizes the AE. It should be noted that bruising at the site of cuff inflation, as well as general discomfort associated with the inflated pressure cuffs, were deemed expected and thus not documented as an AE.

**TABLE 4:**
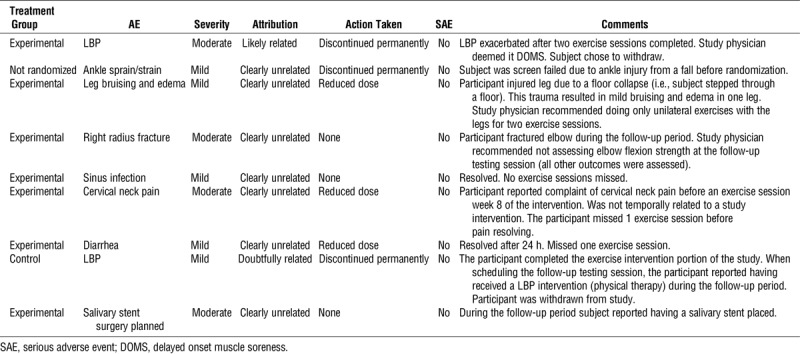
Adverse events.

Average treatment acceptability scores (9–45 scale with a higher score reflective of better treatment acceptance) were relatively high in both groups (BFR exercise group, 31.4 ± 5.7; CON exercise group, 34.5 ± 4.0), and scores were not statistically different between groups (*P* = 0.08).

## DISCUSSION

We found that trunk muscle responses to low-load exercise were not enhanced after BFR exercise of the limb muscles for any of the outcome measures at any time point in this randomized, single-blinded trial. Thus, it appears that attempting to use the cross-transfer effect of BFR exercise is not a viable strategy for enhancing trunk muscle performance while maintaining lower mechanical loading of the spine. More surprising was our observation that BFR did not systematically enhance limb muscle mass and strength to a greater extent than low-load resistance exercise *without* BFR, despite numerous studies with contradictory findings (for reviews and meta-analyses see (2–7). We further discuss these findings below.

The lack of a cross-transfer of effect on the trunk muscles might be a function of an inadequate trunk muscle exercise stimulus. Though the trunk exercise was performed at 25% of isometric maximum, which has been associated with a positive cross-transfer effect (43), it is unknown how much of the isometric maximum or 25% max force was generated by the erector spinae and how much was generated by other muscles (e.g., hip extensors). Thus, the load on the erector spinae during the prescribed exercise protocol could have been insufficient to induce gains in size or strength, even with the addition of BFR, such as previously reported by Abe et al. (43). It could also be that the recurrent, nonspecific LBP population studied here exhibits impaired central activation of the trunk muscles, thus limiting the response to training. However, in our opinion, the most likely explanation for a lack of cross-transfer of effect is a lack of systemic hormonal influence on hypertrophy or strength gains (18,19). Curiously, our most robust finding was an increase in trunk extension strength and endurance in the control group during the follow-up period. We struggle to explain these findings.

As noted, the lack of an effect of BFR on the limb muscles was surprising. Numerous studies over the past 15 to 20 yr have reported that low-load BFR resistance exercise facilitates exercise-induced gains in muscle mass and strength (8,13,39,44,45). Two recent meta-analyses indicated the superiority of BFR resistance training when compared to an equivalent low-load resistance training program without BFR (2,5) and another meta-analysis suggested that low-load blood flow–restricted exercise was equally as effective as high-load resistance exercise at generating muscle hypertrophy (4). Thus, our findings that low-load BFR resistance exercise was no better than low-load resistance exercise is contrary to much of the extant literature. There are a few potential reasons for this.

It could be that the recurrent, nonspecific LBP population had problems in the activation of limb muscles, as individuals with recurrent, nonspecific LBP have been reported to exhibit impairments in quadriceps activation capacity after aerobic exercise (46). It could also be that our exercise protocol differed from many of those performed in previous studies. Here, subjects in both exercise groups were asked to perform the exercise to volitional concentric task failure, while in numerous BFR exercise studies exercise dose has been based on repetition or volume (8,13,39,44,45). Our rationale was to match the groups for volitional effort. Consistent with our findings, Farup and colleagues (47) as well as Fahs and colleagues (48) observed that low-load BFR exercise and low-load resistance exercise without restriction, when performed to volitional failure (three times per week for 6 wk), resulted in equal muscle hypertrophy (47). We would argue that protocols not matching for volitional effort (but rather repetitions or volume) do not directly address the question of whether there is added value to the adaptive response associated with BFR *per se*, but are rather evaluating whether or not low-load exercise when performed to task failure can induce muscle growth and strength gains. With this stated, it should be noted that moderate effect sizes were noted for BFR exercise enhancing muscle size and strength in the leg extensors (*η*^2^ = 0.19 and 0.27, respectively). As such, it is probable that we were underpowered to detect these differences. However, these potentially positive effects (based on effect size interpretation) must be considered alongside the lack of positive effect sizes for BFR exercise in relation to leg extensor muscle endurance as well as muscle strength of the plantar and elbow flexor muscles.

It is also worth noting that our study implemented rigorous control measures that are lacking from much of the previous work on BFR exercise (49,50). For instance, we designed our trial to match for volitional effort (as discussed above), randomized our subjects, and blinded our outcome assessors, data analysts, and statistician. Although we cannot be certain that our discrepant results are due to our bias control measures, it is plausible inasmuch that numerous biases are believed to affect the scientific literature and it has been suggested that these biases are creating crisis (51). Accordingly, we advocate for the inclusion of strong bias controls in future studies and publications in the BFR exercise field.

Lastly, we should note that in the current study both control and BFR exercise groups performed the training at 25% of their maximum strength. Our findings should be interpreted within this context. Specifically, the usage of low-intensity exercise in the appendicular muscles of the control group should be questioned because the limbs have minimal restriction for mechanical load in this scenario. This design does not impact the BFR exercise findings *per se*, but certainly this approach limits the opportunity for the exercise control group to exhibit positive direct and cross-transfer of effect findings.

In conclusion, we did not observe changes in muscle morphology and performance that would support the cross-transfer effect of limb BFR exercise to the TE muscles of individuals with recurrent, nonspecific LBP. Thus, it appears that BFR exercise would provide no added rehabilitative benefit for adults with recurrent, nonspecific LBP. In addition, we did not observe BFR exercise to increase limb muscle outcome measures at any time point. Contrary to prior reports, these findings indicate that BFR exercise does not enhance muscle mass and strength to a greater extent than low-load resistance exercise *without* BFR. These results are contrary to the majority of prior reports on BFR exercise training.

## Supplementary Material

SUPPLEMENTARY MATERIAL
