# Supplementary material for: Blood Flow–restricted Exercise Does Not Induce a Cross-Transfer of Effect: A Randomized Controlled Trial
Source: Med Sci Sports Exerc. 2019 Mar 19;51(9):1817–27. doi: 10.1249/MSS.0000000000001984 (PMC6697223; doi:10.1249/MSS.0000000000001984)
Supplement: SUPPLEMENTARY MATERIAL [file mss-51-1817-s001.doc]

**
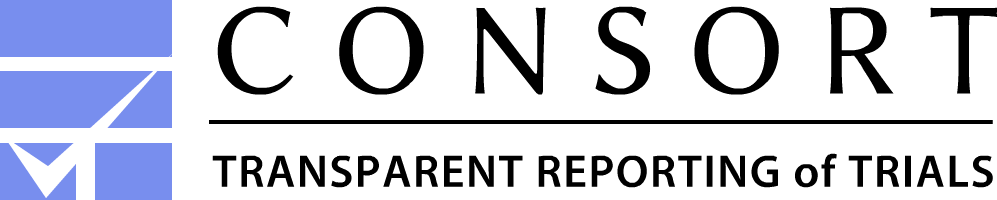
**

**CONSORT Flow Diagram**

**Allocation**

**Analysis**

**Follow-Up**

**Enrollment**

Assessed for eligibility (n= 572)

Excluded (n=540)

♦  Not meeting inclusion criteria (n=537)

♦  Declined to participate (n=3)

Analysed (n=16)
♦ Excluded from analysis (give reasons) (n=0)

Lost to follow-up (n=0)

Discontinued study (n=0)

Allocated to Control Intervention (n=17)

♦ Received allocated intervention (n=16)

♦ Did not receive allocated intervention (participant withdrawn due to exacerbated LBP after baseline testing) (n=1)

Lost to follow-up (n= 0)

Discontinued study (one participant withdrawn due to starting a prohibited intervention during the follow-up period) (n= 1)

Allocated to BFR Intervention (n=15)

♦ Received allocated intervention (n=15)

♦ Did not receive allocated intervention (give reasons) (n=0)

Analysed (n=14)
♦ Excluded from analysis (one participant excluded due to starting a prohibited intervention during the follow-up period) (n=1)

Randomized (n=32)
